# A Prospective Comparative Study Between Ultrasound-Guided Combined Sciatic-Femoral Nerve Block Versus Spinal Anesthesia for the Patients Undergoing Elective Below-Knee Surgeries

**DOI:** 10.7759/cureus.26137

**Published:** 2022-06-20

**Authors:** Bibhuti Pattajoshi, Sanjukta Panigrahi, Premakanta Mohanty, Ranjeet K Mohanty, Sandeep K Panigrahi

**Affiliations:** 1 Anesthesia and Critical Care, Ispat Post-graduate Institute and Superspeciality Hospital, Rourkela, IND; 2 Anesthesia and Critical Care, Ispat General Hospital, Rourkela, IND; 3 General Medicine, Fakir Mohan Medical College and Hospital, Balasore, IND; 4 Anesthesia and Critical Care, Community Welfare Society Hospital, Rourkela, IND; 5 Community Medicine, Institute of Medical Sciences and SUM Hospital, Bhubaneswar, IND

**Keywords:** peripheral nerve block, clinical trial, epidemiological study, adverse effects, pain

## Abstract

Introduction

The scope of anesthesia has shifted from general anesthesia (GA) and spinal anesthesia (SA) for below-knee surgery to peripheral nerve blocks (PNB). Combined sciatic-femoral nerve block (SFNB) with ultrasound (USG) guidance can be a better format for use.

Objectives

The primary objectives were to compare the duration of onset of sensory and motor blockade, total duration of sensory and motor blockade, and time of first analgesic requirement between both groups.

Methods

A prospective, randomized comparative study was carried out at a tertiary care teaching hospital in Odisha, India, from April 2019 to April 2021 in the Department of Anaesthesiology. Patients admitted for elective below-knee surgeries with American Society of Anesthesiology (ASA) grade II or less were divided into two groups (Group A receiving USG-guided SFNB and group B receiving SA) by computer-generated sampling. The block randomization method was used to ensure equal samples in both groups. Data collection was done using the Magpi software (Magpi, Inc., Washington, D.C., United States) on android-based mobile phones. Data were analyzed using Stata Statistical Software: Release 12 (2011; StrataCorp LP, College Station, Texas, United States) for analysis. Relevant statistical tests were used to compare the results between the groups (independent sample t-test or Wilcoxson signed-rank test). Repeated measures ANOVA (RM-ANOVA) was used to check the hemodynamic stability within the groups.

Results

Thirty-seven subjects were enrolled in each arm (Group A and Group B). Baseline parameters in both groups were comparable. The most common indication among the study subjects was single or multiple meta-tarsal fractures (20, 27.0%) followed by malleolus (15, 20.3%) and calcaneum fractures (13, 17.6%). Most of the study subjects were from ASA grade I (around 80%). The time of onset of sensory and motor block was found to be more for USG-guided SFNB (8.08±2.11 minutes and 11.35±1.84 minutes, respectively) as compared to the SA group (3.03±0.50 minutes and 4.89±0.52 minutes, respectively) (p<0.001). Total anesthesia and time to first analgesic requirement were, however, more in USG-guided SFNB (349.43±53.49 minutes and 339.73±54.24 minutes, respectively) as compared to the SA group (137.30±34.21 minutes and 137.30±34.21 minutes, respectively) (p<0.001). The mean time to first urination in USG-guided SFNB (178.92±20.92) was significantly less (p<0.001) compared to the SA group (419.19±40.30).

There were no adverse events (0%) in USG-guided SFNB while 64.9% of the subjects in the SA group experienced adverse events (p<0.001). The most common adverse events were nausea/vomiting and hypotension (around 50% for both). Hemodynamic stability was present in both the groups of anesthesia subjects, though fluctuations in blood pressure may be seen more frequently in cases of SA. All the subjects in both the groups had achieved a Bromage score of 3 universally. The grand mean score of pain by SA (2.347±0.044) was more (p<0.001) in comparison to that in subjects with USG-guided SFNB (1.961±0.073) and this was significant in both the groups. The mean increase in pain score at 24 hours in comparison to baseline was, however, significantly more (p<0.05) in the SA group (1.784±0.111) in comparison to those receiving USG-guided SFNB (1.324±0.190).

Conclusion

USG-guided SFNB is a better option for below-knee surgeries as compared to SA.

## Introduction

Regional anaesthesia and peripheral nerve blocks (PNBs) can help meet the goals of caring for patients in the outpatient setting [1]. Peripheral nerve blocks (PNBs) have similar advantages to neuraxial (spinal and epidural) anaesthetic and analgesic techniques, especially the lack of need for airway instrumentation and hence a priority for subjects with borderline respiratory function. It also allows for shorter discharge times because of fewer possibilities of nausea, vomiting, and severe pain. Chronic pain syndromes are also minimized because of the lack of central nervous system sensitization that occurs after acute injury. Finally, patients with PNB have minimal if any opioid requirements in the immediate postoperative phase [2,3].

Below-knee procedures have become increasingly common due to the effectiveness of the anaesthesia techniques that facilitate rapid and safe discharge [4]. Regional anaesthesia techniques are used as an alternative to general anaesthesia (GA) in these procedures. It is generally accepted that both PNBs and spinal anaesthesia (SA) provide sufficient anaesthesia, and better postoperative analgesia and satisfaction than GA, in addition to being minimally invasive and using fewer resources [5].

SA is particularly preferred by patients undergoing lower limb surgery due to the fact that only the desired region undergoes nerve blockade, which results in relatively early mobilization and good patient satisfaction [6]. PNBs have been used in patients posted for below-knee surgeries and for patients with critical co-morbidities who cannot tolerate even the slightest alteration of hemodynamic status [1,2,7].

PNBs provide surgical anaesthesia with better cardiorespiratory stability as compared to the SA blockade which has side effects of hypotension, bradycardia, meningitis, postdural puncture headache, hematoma, and neurological deficit, etc. With the development of new techniques such as ultrasound (USG), the scope of anaesthesia has shifted from GA and SA for below-knee surgery to PNBs [2,3].

Combined sciatic-femoral nerve block (SFNB) without USG guidance can also be used for below-knee surgeries, but it remains a less frequently used technique as compared to USG-guided because it may take a longer time to perform, incurs multiple pricks, has failures due to its anatomical position and variation, requires a higher dosage of local anaesthetic, and may cause postoperative paraesthesia [8]. USG-guided combined SFNB thus remains the preferred technique because it avoids landmark-guided SFNB-related side effects and is associated with a number of benefits such as less needle insertion, improved block quality [9], short administration time, decreased dosage of local anaesthetic, and rapid onset of nerve blockade [7,8].

A prospective comparative trial was conducted to observe and compare USG-guided SFNB and SA for patients undergoing all kinds of elective below-knee surgeries as no such studies existed at the time point of conducting the study.

Objective

The objective of this study is to compare the time of onset of sensory and motor blockade, total duration of sensory and motor blockade, and time of first analgesic requirement between USG-guided SFNB and SA for patients undergoing elective below-knee surgeries.

## Materials and methods

The study was a prospective, randomized comparative study (Level II evidence, a little lower in the level of evidence than the classic randomized control trial (RCT)). It was carried out in the Department of Anaesthesiology of Ispat General Hospital, Rourkela, in Western Odisha, India. The study, including protocol and tool development, ethical approval, data collection, analysis, and reporting was conducted from April 2019 to April 2021. The trial was also registered prospectively at Clinical Trials Registry- India (CTRI) website on December 29, 2020 (registration number CTRI/2020/12/030090). Recruitment and data collection began after registering at CTRI. Patients admitted to the hospital for elective below-knee surgeries were asked for consent. Of the patients who gave consent, those who met the inclusion and exclusion criteria were enrolled in the study. Inclusion criteria included age between 16 to 65 years, American Society of Anaesthesiologists (ASA) grade I and II, and patients scheduled for elective below-knee surgeries and had filled up the consent form to undergo surgery and anaesthesia process. Patients who were allergic to local anaesthetics, had bleeding disorders, localised infections, neurological disease, anatomical abnormalities of the spinal column, respiratory or cardiac disease, anterior cruciate ligament tear, and body mass index (BMI) > 32 kg/m2 were excluded from the study.

Patients were randomly divided into two groups by computer-generated sampling. To ensure equal samples in each group, the block randomization method was used. Group A was for USG-guided combined SFNB while group B was for SA.

The sample size was estimated considering two important facts: (i) effective sample size when considering the difference between the two groups with change in time, and (ii) the availability of subjects in the stipulated timeline for the study (since it was academic in nature). 

For considering an effective estimation of difference between the two groups using repeated measures ANOVA (RM-ANOVA) test with an alpha error of 0.05, power of the study 0.9, number of measurements as 19 (as per the number of time points during which observations were to be recorded), and effect size as 0.25 the total estimated.

As per admission prevalence in the hospital, the sample size was calculated using the formula:

(n = (t^2 x p (1-p))/m^2)​

where, n = required sample size; t = confidence level at 95% (standard value of 1.96); p = estimated prevalence of below-knee surgery in the area = 5% = 0.05; m = margin of error at 5% (standard value of 0.05) = 0.05.

Substituting the above-mentioned values in the formula, we get 72.96 as the required sample, which was rounded to 74 for the convenience of dividing into two equal groups or 37 in each group. This sample size was near about the same levels that were required as per RM-ANOVA calculation and was the final sample that was used as per feasibility for the study. It was decided that post-hoc estimate of power would be done based on mean levels of the total time of anaesthesia in the groups and pooled standard deviation, as per the number of samples finally achieved.

In the USG-guided combined SFNB group (group A), the sciatic and femoral nerve blocks were achieved using a 25 ml mixture consisting of 10 ml of lignocaine adrenaline, 10 ml of 0.5% bupivacaine, and 5ml of saline (10ml for the femoral and 15 ml for the sciatic nerve block) under USG guidance using MyLab™ One (Esaote SpA, Genoa, Italy). In group B, SA was achieved by injecting 2 ml (10 mg) of 0.5% bupivacaine (hyperbaric) under all aseptic precautions at the lumbar L3-L4 level through a 25-gauge Quincke spinal needle (Becton, Dickinson, and Company (BD®), Franklin Lakes, New Jersey, United States) in the sitting upright position and median approach.

All the nerve blocks were performed by the Principal Investigator (PI) or Guide. The PI was trained on the technique of anaesthesia and his technique was validated by the Guide before the study. The onset of the block was evaluated in the operating theatre for each from one minute to 30 minutes. If a complete block was not achieved in PNB, it was considered a failed block, and the subject was excluded from the study to avoid bias, but since ethically the subject must be operated on, they were operated under GA/SA.

The endpoint of the study was the timing of rescue analgesia or 24 hours, whichever was earlier. If Visual Analog Score (VAS) was >4 then rescue analgesia in the form of inj. diclofenac 1-1.5mg/kg iv was administered. Postoperative analgesia used was IV paracetamol 20 mg/kg eight hourly and when VAS ≥ 4, rescue analgesia was provided with intermittent boluses of inj. diclofenac 1-1.5mg/kg iv. Time to the first rescue analgesic request and supplemental rescue analgesic requirements, if any, were recorded over 24 hours by the investigator. Postoperative pain was evaluated by VAS. Hemodynamic parameters (blood pressure and heart rate) and side effects were noted in all patients at time points 0, 1, 2, 4, 8, 12, and 24 hours postoperatively.

The tool for data collection was based on the objectives and investigations routinely done in the setup where the study was conducted. It included time of admission and time of discharge, age, weight, ASA score, pre-operative investigations such as haemoglobin levels, complete blood count, bleeding and clotting time, random blood sugar, blood urea, serum creatinine, serum sodium, serum potassium, electrocardiogram, chest x-ray, and other relevant investigations. The primary outcome measure in this study such as the time to first rescue analgesic request and the total cumulative requirements of rescue analgesic during 24 hours postoperative study period between the two groups were captured using the tool. The secondary outcome measures include assessment of pain scores (VAS), haemo-dynamics (every five minutes till 30 minutes, every 10 minutes till 90 minutes, and then at 2, 4, 6, 12, and 24 hours), and side effects. Grading of sensory and motor blockade was also recorded on a Likert scale with the time of onset of the same. Along with this, total anaesthesia time was also noted. VAS from 0 to 10 was used to note the intensity of pain (1 hour, 2 hours, 4 hours, 6 hours, 12 hours, and 24 hours).

The tool for data collection was entirely developed on Magpi software (Magpi, Inc., Washington, D.C., United States), after registering as a basic user [10]. Mobile-based data collection tool was made available that could be used both online and offline through the Magpi software, and data collection was done simultaneously on a real-time basis. Since the number of variables exceeded the limit meant for Magpi basic users, two tools were made, and a unique identifier (registration number) was used later to merge the data sheet.

Data was downloaded by importing it from the online software into Excel sheets (Microsoft Corporation, Redmond, Washington, United States). Two such Excel sheets were downloaded containing all the variables for the study. The data were then merged using unique identifiers (registration number of the subject) and then compiled and cleaned before importing to Stata Statistical Software: Release 12 (2011; StrataCorp LP, College Station, Texas, United States) for analysis [11]. Data were described using mean and standard deviation for quantitative data and numbers with percentages for qualitative data. A comparison was done for variables that defined the objectives, between the groups, after understanding the nature of distribution using relevant statistical tests. Parametric tests (independent sample t-test) were used for analysing normally distributed data, while non-parametric test (Wilcoxson signed-rank test) was used to compare the variables that were not normally distributed (time to sensory blockade, time to motor blockade, time to anaesthesia, time to requirement of analgesic, etc.). RM-ANOVA [12] was used to compare the variability of the hemodynamic measures within the groups over the period of time of observation.

Ethical clearance

Ethical clearance for the study was taken from the Institutional Review Board (Ethical Committee) of Ispat General Hospital, Rourkela, Odisha, India (approval letter number IEC/IGH/2019/8, dated April 23, 2019). An informed consent form (ICF) which included a patient information sheet (PIS) was used for taking the consent from the subject in bilingual language (English/Odia). The Odia ICF used was a translated approved version of the English ICF and was used for subjects who were not able to understand or read and write in English. Confidentiality was maintained at all stages. Possible adverse events (solicited) were explained to the subjects before taking the consent. All queries of the subjects were carefully addressed to their satisfaction before signing the consent form by the subject and the PI. All steps of the consent process were followed as per good clinical practice (GCP) rules. Data used and exported for analysis were coded without revealing the subject's name and consisted of only the initials of the subject and subject identification code.

## Results

The total number of subjects enrolled in the study was 74, with 37 subjects in each arm (Group A and Group B). Matching with respect to age, gender, and BMI was ensured and confirmed through baseline parameter comparison. There were 49 male subjects as compared to 25 female subjects in the study. The distribution of gender within the groups was almost equal with no significant difference in between the groups (p>0.05). The mean age of the study subjects was found to be 46.13 ± 10.23 years in group A and 42.30 ± 7.64 years in group B (44.22 ± 9.18 years in total). However, the distribution of age within the groups was found to be non-normal (p<0.05, Shapiro Wilk Normality test). Age-wise comparison of the median values showed that there was no statistically significant difference between the groups (p=0.064, Mann-Whitney U test). Similar was the case for BMI, which was comparable in both groups (p>0.05) (Table 1). Thus, age and BMI were the basic parameters that were comparable at baseline, ensuring proper randomization.

**Table 1:** Comparison of baseline parameters in the study subjects between the groups (n=74; 37 in each group) † Normally distributed data, compared using independent sample t-test; rest were compared using Mann-Whitney U test *Statistically significant at p<0.05 **Statistically significant at p<0.01 Gr A=USG-guided sciatic-femoral nerve block group; Gr B=Spinal anaesthesia group IQR: interquartile range; SpO2: oxygen saturation; P50: haemoglobin-oxygen affinity

Parameter	Group	Mean	SD	P50	IQR	p-value
Body mass index (kg/m^2^)	Gr A	24.08	1.59	24.34	2.49	0.3413
	Gr B	24.54	2.88	24.68	2.78	
Haemoglobin (mg/dl)^ †^	Gr A	12.77	1.47	12.60	2.10	0.515
	Gr B	12.99	1.37	13.30	1.80	
Heart rate (beats/ min)	Gr A	76.14	8.62	72.00	10.00	0.3364
	Gr B	76.59	5.65	78.00	10.00	
Systolic blood pressure (mm Hg)^ †^	Gr A	131.11	14.19	130.00	20.00	0.04*
	Gr B	136.92	9.78	140.00	14.00	
Diastolic blood pressure (mm Hg)^ †^	Gr A	77.71	9.15	78.00	8.00	0.0031**
	Gr B	83.68	7.54	82.00	10.00	
Mean arterial pressure (mm Hg)	Gr A	95.50	10.09	93.33	10.67	0.0028**
	Gr B	101.42	7.22	101.33	8.00	
SpO_2_ (%)	Gr A	99.57	0.60	100.00	1.00	<0.001**
	Gr B	98.97	0.50	99.00	0.00	

Hemodynamic parameters were assessed for both the groups at baseline (Table 1). Systolic blood pressure (SBP), diastolic blood pressure (DBP), and haemoglobin values showed normal distribution in both groups (p>0.05). Heart rate, mean arterial pressure, and oxygen saturation, however, were found to have non-normal distribution (p<0.05) in one or both groups. An appropriate statistical test was used (as given in the legend of Table 1) based on the distribution of data. A comparison of baseline parameters between the groups showed that systolic, diastolic, and mean arterial pressure were significantly more in group B (SA) at baseline as compared to group A (USG-guided combined SFNB). Oxygen saturation levels, however, were more in group A as compared to group B and this was statistically significant (p<0.01) (Table 1).

The most common indication among the study subjects was single or multiple meta-tarsal fractures (n=20, 27.0%) followed by malleolus (15, 20.3%) and calcaneum fractures (13, 17.6%). Apart from various kinds of below-knee fractures, a few subjects in the study also underwent split skin grafts (5, 6.8%) (Table 2).

**Table 2:** Indications for operations among subjects in the study by the group of anaesthesia (n=37 in each group) Group A=USG-guided sciatic-femoral nerve block group; Group B=Spinal anaesthesia group

Indication	Group A n (%)	Group B n (%)	Total n (%)
Metatarsal fracture	12 (32.4)	8 (21.6)	20 (27.0)
Malleolar fracture	8 (21.6)	7 (18.9)	15 (20.3)
Calcaneum fracture	8 (21.6)	5 (13.5)	13 (17.6)
Split skin graft	2 (5.4)	3 (8.1)	5 (6.8)
Tibial fracture	2 (5.4)	3 (8.1)	5 (6.8)
Fibula fracture	2 (5.4)	1 (2.7)	3 (4.1)
Others	3 (8.1)	10 (27.0)	13 (17.6)
Total	37 (100.0%)	37 (100.0%)	74 (100.0%)

The subjects in the study group mostly belonged to ASA grade II or less. There was no category of subjects with ASA grade III and above. The majority of the subjects belonged to ASA grade I (59 cases, 79.7%) (Table 3). 

**Table 3:** American Society of Anesthesiologists (ASA) grading of the study subjects (n=37 in each group) ^ four cases (10.8%) of Group A included in Grade II had systemic disease but not incapacitating

ASA Grade	Group A n (%)	Group B n (%)	Total n (%)
I	24 (64.9)	35 (94.6)	59 (79.7)
II^	13 (35.1)	2 (5.4)	15 (20.3)
III and more than III	0 (0)	0 (0)	0 (0)
Total	37 (100.0)	37 (100.0)	74 (100.0)

Time taken for onset of sensory blockade, motor blockade, total anaesthesia time, time to analgesic requirement and time to first spontaneous urination were checked for normality. It was seen that all the variables were normally distributed within the groups (p>0.05; Shapiro-Wilk normality test). The time of onset of sensory and motor block was found to be more for group A (8.08±2.11 minutes and 11.35±1.84 minutes, respectively) as compared to group B (3.03±0.50 minutes and 4.89±0.52 minutes, respectively), and this was found to be statistically significant (p<0.001; independent sample t-test). However, the average time of total anaesthesia and time to first analgesic requirement (the point beyond which subjects could not resist pain and opted for analgesia) were more in group A (349.43±53.49 minutes and 339.73±54.24 minutes, respectively) as compared to group B (137.30±34.21 minutes and 137.30±34.21 minutes, respectively), and this was found to be statistically significant (p<0.001; independent sample t-test) (Table 4). The mean time to first urination in group A (178.92±20.92) was significantly less (p<0.001; independent sample t-test) compared to group B (419.19±40.30).

**Table 4:** Comparison of various time points after anaesthesia between the study groups (n=74, 37 in each group) †Independent sample t-test were applied as data was normally distributed within the groups **Statistically significant at p=0.001 levels Group A=USG-guided sciatic-femoral nerve block group; Group B=Spinal anaesthesia

Parameter†	Group	Mean	SD	P50	IQR	p-value
Time to onset of sensory blockade (in mins)	Gr A	8.08	2.11	8	4	<0.001**
	Gr B	3.03	0.50	3	0	
Time to onset of motor blockade (in mins)	Gr A	11.35	1.84	12	3	<0.001**
	Gr B	4.89	0.52	5	0	
Total anaesthesia time (in mins)	Gr A	339.43	53.49	360	60	<0.001**
	Gr B	137.30	34.21	130	30	
Total time to analgesic requirement (in mins)	Gr A	339.73	54.24	360	60	<0.001**
	Gr B	137.30	34.21	130	30	
Time to first urination (in mins)	Gr A	178.92	20.92	180	30	<0.001**
	Gr B	419.19	40.30	420	60	

There were no adverse events in the study subjects after anaesthesia in Group A (0%) while 24 subjects in Group B experienced adverse events (64.9%). The most common adverse events were nausea/vomiting and hypotension (19, 51.4%) with similar incidences in both. Headache was also seen as an adverse event in a few cases (6, 16.2%). There was a significant association of SA with the presence of adverse events (c2=35.52, p<0.001) (Table 5).

**Table 5:** Comparison of adverse events after anaesthesia between the study groups (n=74, 37 in each group) Group A=USG guided SFNB; Group B=Spinal Anaesthesia

Adverse event type	Group A n (%)	Group B n (%)	Chi-square	p-value
Pain	0 (0%)	0 (0%)	-	-
Nausea/ Vomiting	0 (0%)	19 (51.4%)	22.944	<0.001
Headache	0 (0%)	6 (16.2%)	4.534	0.033
Drowsiness	0 (0%)	0 (0%)	-	-
Hypotension	0 (0%)	19 (51.4%)	22.944	<0.001
Arrhythmia	0 (0%)	0 (0%)	-	-
Hypertension	0 (0%)	0 (0%)	-	-
Hypoxia	0 (0%)	0 (0%)	-	-
Total	0 (0%)	24 (64.9%)	35.520	<0.001

**Figure 1:**
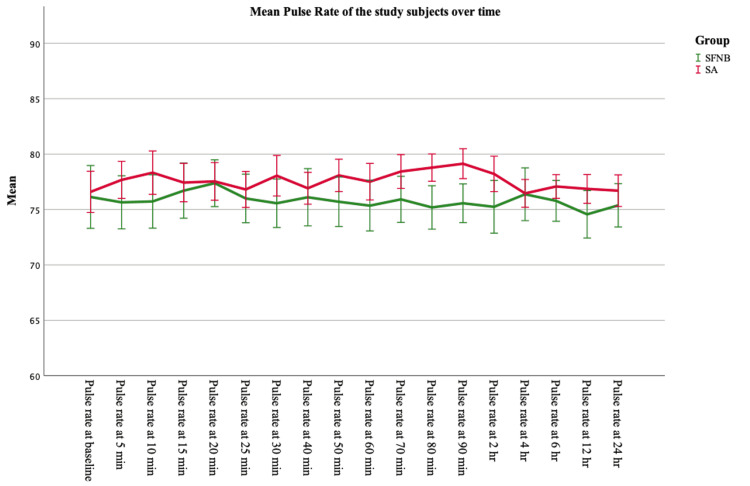
Comparison of trend in pulse rate between the study groups (n=74, 37 in each group) Error Bars: 95% Confidence interval SFNB: sciatic-femoral nerve block; SA: spinal anaesthesia

**Figure 2:**
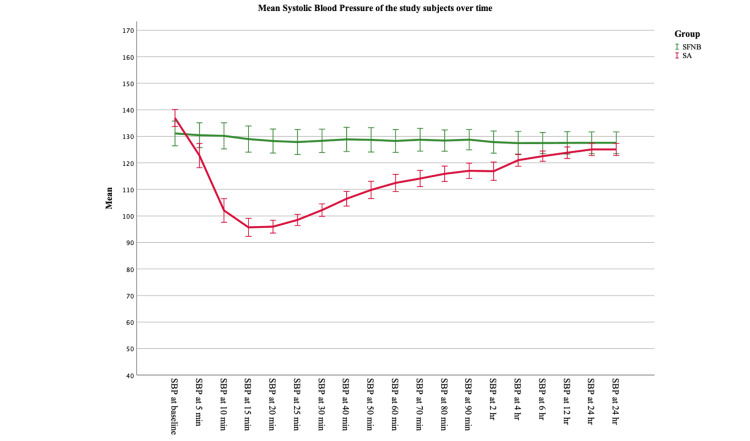
Comparison of trend in systolic blood pressure between the study groups (n=37 in each group) Error bars: 95% Confidence interval SBP: systolic blood pressure; SFNB: sciatic-femoral nerve block; SA: spinal anaesthesia

**Figure 3:**
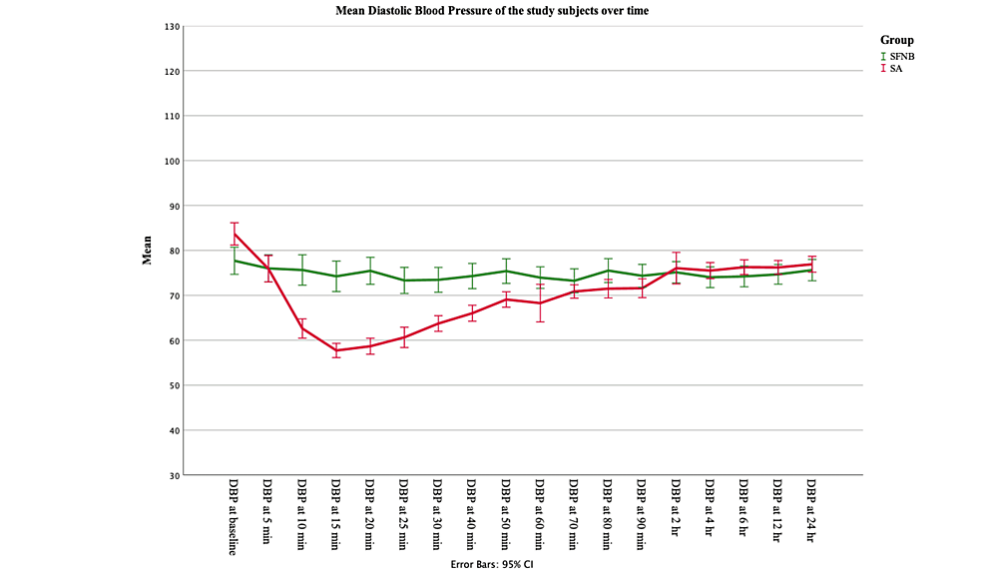
Comparison in trend of diastolic blood pressure between the study groups (n=37 in each group) Error bars: 95% Confidence interval DBP: diastolic blood pressure; SFNB: sciatic-femoral nerve block; SA: spinal anaesthesia

The mean heart rate in the intra-operative period of Group B subjects (77.59) was found to be slightly higher as compared to group A (75.80) after anaesthesia. However, SBP and DBP were found to be higher in Group A subjects (SBP 128.6 and DBP 74.8) as compared to Group B (SBP 113.3 and DBP 70.1). Oxygen saturation levels were almost similar in both groups (Table 6). The haemodynamic parameters were compared for within-subject variation with time using RM-ANOVA. The data were tested for sphericity using Mauchly’s sphericity test, and it was found to be significant for all values in both groups (p<0.05). Hence, the Greenhouse-Geisser test was used to find if there was any significant difference among the subjects with respect to different time points. It was seen that heart rate and oxygen saturation were not significantly different with time, in both the groups (p>0.05). SBP and DBP were found to be significantly different with time in both the groups (p<0.05), though the significance was at a very high level for subjects undergoing spinal anaesthesia (p<0.001) as compared to subjects undergoing USG-guided SFNB (p<0.05) (Table 6, Figures 1, 2, 3).

**Table 6:** Haemodynamic parameters after anaesthesia between the study groups (n=74, 37 in each group) p-value is calculated at 0.05 levels using RM-ANOVA (Wilk’s lambda) incorporating within-subject variation by 18 time points † grand mean with standard error (SE); ‡ Using Greenhouse-Geisser test as Mauchly’s test of sphericity is significant and sphericity is not assumed Group A=USG-guided sciatic-femoral nerve block group; Group B=Spinal anaesthesia SBP: systolic blood pressure; DBP: diastolic blood pressure; SpO2: oxygen saturation; RM-ANOVA: repeated measures analysis of variance

Parameter	Mean †	SE †	F-statistics ‡	p-value
Heart rate				
Group A	75.80	0.96	1.059	0.393
Group B	77.59	0.46	1.532	0.132
SBP				
Group A	128.60	2.12	2.656	0.018
Group B	113.29	0.97	91.356	<0.001
DBP				
Group A	74.79	1.91	2.655	0.005
Group B	70.07	0.46	47.447	<0.001
SpO_2_				
Group A	99.58	0.067	0.608	0.774
Group B	99.89	0.092	1.015	0.423

Assessment of motor blockade using Bromage score revealed that 100% of the subjects in both the scores had achieved a Bromage score of 3 universally. Assessment of pain was done in the preoperative period (baseline) and at intervals of 1 hour, 2 hours, 4 hours, 6 hours, 12 hours, and 24 hours. Change in VAS with time was assessed using RM-ANOVA while the comparison of mean changes between the groups (at 24 hours from baseline) was assessed using Independent sample t-test. It was seen that the grand mean score of pain by SA (2.347±0.044) was more in comparison to that in subjects with USG-guided SFNB (1.961±0.073) (Table 7). However, the change with time observed was significant in both groups (p<0.001). The mean increase in VAS at 24 hours in comparison to baseline was, however, significantly more (p<0.05) in SA group of subjects (1.784±0.111) in comparison to those receiving USG-guided SFNB (1.324±0.190).

**Table 7:** Pain during anaesthesia between the study groups (n=74, 37 in each group) **p-value is calculated at 0.05 levels using RM-ANOVA (Wilk’s lambda) incorporating within-subject variation by 18 time points † Grand mean with standard error (SE); ‡ Using Greenhouse-Geisser test as Mauchly’s test of sphericity is significant and sphericity is not assumed Group A=USG-guided sciatic-femoral nerve block group; Group B=Spinal anaesthesia VAS: Visual Analogue Scale for assessment of pain; RM-ANOVA: repeated measures analysis of variance

Parameter	Mean †	SE †	F-statistics ‡	p-value
VAS				
Group A	1.961	0.073	64.074**	<0.001
Group B	2.347	0.044	202.327**	<0.001

## Discussion

The study was a prospective randomized comparative study. Interventions that were used in the study groups for anaesthesia were routinely used at the institute where the study was conducted.

A total of 74 subjects were randomly allocated to either group of intervention and the anaesthetic and operative procedures were completed as per plan and protocol followed in the hospital. There was no significant difference in gender between the groups selected for anaesthesia (p>0.05). The mean age of the study subjects was found to be 46.13 ± 10.23 years in group A and 42.30 ± 7.64 years in group B (44.22 ± 9.18 years in total), and there was no significant difference in the mean age between the groups (p>0.05). This proves that bias during selection was absent for age and gender and both the groups were comparable with respect to age and gender.

Comparison of baseline parameters between the groups showed that SBP, DBP, and mean arterial pressure were significantly more in group B (SA group) at baseline as compared to group A (USG-guided combined SFNB). Oxygen saturation levels, however, were more in group A as compared to group B and this was statistically significant (p<0.01). These parameters got adjusted during RM-ANOVA analysis while considering changes with time.

The most common indication among the study subjects was single or multiple meta-tarsal fractures (20, 27.0%) followed by malleolus (15, 20.3%) and calcaneum fractures (13, 17.6%). Apart from various kinds of below-knee fractures, a few subjects in the study also underwent split skin grafts (5, 6.8%). The majority of the subjects belonged to ASA grade I (59 cases, 79.7%).

The time of onset of sensory and motor block was found to be more for USG-guided SFNB (8.08±2.11 minutes and 11.35±1.84 minutes respectively) as compared to the SA group (3.03±0.50 minutes and 4.89±0.52 minutes respectively), and this was found to be statistically significant (p<0.001; independent sample t-test). Total anaesthesia and time to first analgesic requirement were, however, more in USG-guided SFNB (349.43±53.49 minutes and 339.73±54.24 minutes, respectively) as compared to the SA group (137.30±34.21 minutes and 137.30±34.21 minutes, respectively), and this was found to be statistically significant (p<0.001; independent sample t-test). The mean time to first urination in USG-guided combined SFNB (178.92±20.92) was significantly less (p<0.001; independent sample t-test) compared to the SA group (419.19±40.30). Thus, USG-guided combined SFNB takes more time for onset, but the duration of anaesthesia and even recovery are better as compared to spinal anaesthesia.

In the study conducted by Karaduman et al. [13] in 2020 in Turkey, 60 patients aged 18-65 years were randomly divided into two groups receiving SA and SFNB. The duration of the intervention, time to onset of sensorial and motor block, time to start of surgery, motor block reversal time, and time to first postoperative analgesic were significantly longer in the SFNB group. Fewer patients required rescue analgesia in the first postoperative 24 hours compared with the SA group.

Oberndorfer et al. [9] conducted a randomized trial in 2007 in South Africa. Forty-six children were randomized to receive sciatic and femoral nerve blocks under either USG or nerve stimulator guidance. The duration of analgesia was longer in the USG group; mean and SD of 508 and 178 minutes versus 335 and 169 minutes. The volume of local anaesthetic in sciatic and femoral nerve blocks was reduced with USG compared with nerve stimulator guidance (0.2 ± 0.06 versus 0.3 ml/kg and 0.15 ± 0.04 versus 0.3 ml/kg).

Adverse events after anaesthesia were absent in USG-guided SFNB study subjects (0%) while there were 24 subjects in the SA group who experienced adverse events (64.9%). The most common adverse events were nausea/ vomiting and hypotension (19, 51.4%) with similar incidences in both. Headache was also seen as an adverse event in a few cases (6, 16.2%). There was a significant association of spinal anaesthesia with the presence of adverse events ($\chi ^{2}$=35.52, p<0.001). These adverse events seen in the SA group are the solicited ones.

Heart rate and oxygen saturation were not significantly different with time in both groups (p>0.05). SBP and DBP were found to be significantly different with time in both the groups (p<0.05), though the significance was at a very high level for subjects undergoing spinal anaesthesia (p<0.001) as compared to subjects undergoing USG-guided SFNB (p<0.05). Thus, hemodynamic stability was present in both the groups of anaesthesia subjects, though fluctuations in blood pressure may be seen more frequently in cases of SA.

The incidence and nature of complications in the SA groups in the current study are similar to the study conducted by Akkaya et al. [14] comparing 15 receiving blocks with 15 receiving SA in Turkey in 2014. It showed that the arterial pressure was signifi­cantly lower and the incidence of nausea was higher in the SA. Saturation and patient satisfaction were lower in the block group, while the numerical pain score and the Ramsay sedation score were higher. The results regarding complications were also similar to Palkhiwala et al. [15] study conducted in 2015 in Ahmedabad where none of the patients had any cardiovascular or neurological complications as in the current study. SFNB proved to be a safe method even in severe comorbidities in the study by Tantry et al. [16] in 2010 in Mangalore. They used combined sciatic and femoral nerve block for lower limb surgery in two patients with severe cardiac valvular lesions. It was seen that after 20 minutes of the sciatic block, the patients were totally relieved of pain. Complete sensory and motor recovery was achieved after nine hours.

Assessment of motor blockade using Bromage score revealed that 100% of the subjects in both the scores had achieved a Bromage score of 3 universally. The results were better as compared to Palkhiwala B et. al. [15] study conducted in 2015 in Ahmedabad to observe the outcomes in combined femoral and sciatic nerve block where complete block was achieved in only 92% of the patients.

Assessment of pain was done during the preoperative period (baseline) and at intervals of 1 hour, 2 hours, 4 hours, 6 hours, 12 hours, and 24 hours. It was seen that the grand mean score of pain by SA (2.347±0.044) was more in comparison to that in subjects with USG-guided SFNB (1.961±0.073). However, the change with time observed was significant in both groups (p<0.001). The mean increase in VAS at 24 hours in comparison to baseline was, however, significantly more (p<0.05) in the SA group of subjects (1.784±0.111) in comparison to those receiving USG-guided SFNB (1.324±0.190).

In the study by Khanna et al. [17] in 2021 in Maharashtra to observe the results in PNB, a total of 41 patients participated using femoral, lateral cutaneous femoral nerve, obturator, and sciatic nerve block (FLOS). Results showed there was less requirement for opioids in this technique. FLOS block provided stable hemodynamic, prolonged, and better postoperative analgesia. Intraoperative anaesthesia was very satisfactory for surgery. The requirement of total doses of rescue analgesics, VAS score preoperatively for 48 hours, the incidence of side effects, and complications were very few. This result was almost similar to the present study.

Post hoc assessment of power was done based on mean and pooled standard deviation of the total time of anaesthesia for the study (mean(A)=339, mean(B)=137, pooled SD=44.8, number of measurements as per time points=19, number of groups=2, total sample size=74) for RM-ANOVA analysis of difference between the groups, and power was found to be 1.00. This result validated that a sufficient sample size was used for the study.

## Conclusions

USG-guided SFNB requires a greater time for onset of sensory and motor blockade as compared to SA. However, the total anaesthesia time and time to the first analgesic requirement in the case of USG-guided SFNB is significantly more as compared to SA. Mean time to first spontaneous urination is also significantly less in cases of USG-guided SFNB as compared to SA. The adverse events in the study subjects after anaesthesia were absent in USG-guided SFNB but were present in the SA group. Haemodynamic fluctuations can be seen in both groups, though blood pressure fluctuation is expected to be more in the SA group. Similarly, mean increase in pain score as compared to baseline is significantly more in the SA group as compared to the USG-guided SFNB group.

Considering all the evidence from the current study, it can be said that USG-guided SFNB is a better option for below-knee surgeries as compared to SA.
